# Association between a publicly funded universal drug program and antipsychotic and antidepressant medication dispensing to children

**DOI:** 10.1186/s12887-024-05345-2

**Published:** 2025-02-08

**Authors:** Sophie A. Kitchen, Tara Gomes, Mina Tadrous, Kathleen Pajer, William Gardner, Yona Lunsky, Melanie Penner, David Juurlink, Muhammad Mamdani, Tony Antoniou

**Affiliations:** 1 ICES, Toronto, Canada; 2 Unity Health Toronto, Toronto, ON Canada; 3 Leslie Dan Faculty of Pharmacy, University of Toronto, Toronto, ON Canada; 4 Institute of Health Policy, Management & Evaluation, University of Toronto, Toronto, Canada; 5 Women’s College Hospital, Toronto, ON Canada; 6 Department of Psychiatry, University of Ottawa, Ottawa, ON Canada; 7 CHEO Research Institute, School of Epidemiology & Public Health, University of Ottawa, Ottawa, ON Canada; 8 Centre for Addiction and Mental Health, Toronto, Canada; 9 Autism Research Centre, Bloorview Research Institute, Holland Bloorview Kids Rehabilitation Hospital, Toronto, Canada; 10 Department of Pediatrics, University of Toronto, Toronto, ON Canada; 11 Department of Medicine, University of Toronto, Toronto, ON Canada; 12 Temerty Faculty of Medicine, University of Toronto, Toronto, ON Canada; 13 Dalla Lana Faculty of Public Health, University of Toronto, Toronto, ON Canada; 14 Vector Institute, Toronto, ON Canada

**Keywords:** Antidepressant, Antipsychotic agents, Child, Adolescent, Time-series analysis, Policy analysis

## Abstract

**Background:**

The prescribing of antidepressants and antipsychotics to children has increased worldwide, but little is known about how changes in drug funding policy influence the practice. In 2018, Ontario introduced a universal pharmacare program (OHIP+) for children and youth, amending it in April 2019 to cover only those without private insurance. We examined the association of these policy changes with antipsychotic and antidepressant medication prescribing.

**Methods:**

We conducted a population-based study of antidepressant and antipsychotic medication dispensing to children ≤ 18 years old between September 1, 2014, and February 29, 2020. We obtained dispensing data from the IQVIA Geographic Prescription Monitor database, and used interventional autoregressive integrated moving average models to examine whether the implementation of OHIP + and its subsequent revision were associated with changes in dispensing.

**Results:**

The implementation of OHIP + was not associated with changes in the rate of antidepressants (-19.3 units per 1,000 population; 95% confidence interval [CI]: -41.7 to 3.1) or antipsychotics (+ 1.0 unit per 1,000 population; 95% CI: -5.4 to 7.5) dispensed. Similarly, subsequent changes to the program restricting coverage to children without private insurance were not associated with antidepressant (0.3 units per 1,000; 95% CI: -7.4 to 7.9) or antipsychotic (1.0 units per 1,000; 95% CI: -0.9 to 2.9) dispensing trends.

**Conclusion:**

Implementation of a publicly-funded pharmacare program did not influence trends in antidepressant or antipsychotic medication dispensing among children.

## Introduction

Mental health conditions are common in children and youth. Specifically, the six-month prevalence of any anxiety, mood or behavioural disorder among children aged 4 to 17 years in Ontario, Canada ranged from 18 to 22% in 2014 [1]. In addition to being associated with poor educational and employment outcomes, mental health conditions account for nearly one in four hospital admissions for Canadian children and youth aged 5 to 24 years [2, 3]. Moreover, despite being relatively rare, schizophrenia is the third most common reason for hospitalizations among children and youth with mental health conditions [3, 4]. Antidepressants and antipsychotics can improve symptoms of these conditions, and help children and youth remain in their homes and schools [3, 5–7]. However, few of these drugs have regulatory approval for the treatment of mood disorders in children. Moreover, although antipsychotics are indicated for the treatment of schizophrenia and bipolar disorder, these drugs are commonly used off-label for the management of non-psychotic disorders and externalizing symptoms in children and youth [8, 9]. In addition, antidepressants and antipsychotics have been associated with harm in children and youth. Specifically, antipsychotics can cause metabolic disturbances and weight gain, increasing the risk of early-onset type 2 diabetes, cardiovascular disease, and sudden death [10–12]. Antidepressants have been associated with an increased risk of cardiac events and suicidal thoughts [5]. Balancing access to antidepressant and antipsychotic therapy against the potential for harm associated with the widespread off-label use of these medications is needed to optimize the management of mental health conditions in children and youth.

Several factors have been previously identified as influencing antidepressant and antipsychotic use in children, including patient sex, socioeconomic status and comorbidities [13–17]. Specifically, antidepressants are more commonly prescribed to females, whereas antipsychotic use is higher among males [13–16]. In addition, low-income children are more likely to be prescribed antipsychotics than high-income children, with one Ontario study finding that the same physician was more likely to prescribe these drugs to a child from a low-income family relative to a high-income child with the same medical history [17]. Furthermore, low-income children were less likely to be prescribed serotonin reuptake inhibitors [17]. Prescription drug insurance status may represent another important determinant of access to antidepressants and antipsychotics for children. However, despite residents of Ontario, Canada having publicly-funded health insurance covering the costs of physician and hospital services, prescription drug costs have historically been covered only for families receiving social assistance and/or disability income supports and those aged 65 years and older.

On January 1, 2018, the province of Ontario implemented a universal prescription drug coverage plan called OHIP + for children and youth aged 24 years and younger [18]. OHIP + provided all children and youth aged 24 and under access to all prescription medications listed on the Ontario Drug Benefit formulary at no cost. The OHIP + program was developed to provide all Ontario children and youth aged 24 and under with universal, publicly funded drug coverage, thereby removing out-of-pocket medication costs as a barrier to medication access in this population. The program was subsequently modified in April 2019 to apply only to those without private insurance [19]. These successive policy changes provide a natural experiment for studying the implications of changes in prescription coverage on antidepressant and antipsychotic use among children and youth. While this policy may facilitate access to pharmacotherapy, the possibility exists that removing cost as a barrier could promote overprescribing of these medications. Accordingly, our objective was to examine the association of universal drug coverage with antidepressant and antipsychotic medication dispensing trends to children 18 years of age and under in Ontario, home to approximately 40% of Canadian children [20]. Our specific research question was whether antidepressant and antipsychotic use would increase during the period of publicly funded pharmacare. We focused specifically on antipsychotics and antidepressants because prescribing of these drugs has increased to children and adolescents over time, despite the lack of regulatory approval and safety concerns [21–24]. We also focused specifically on Ontario because it was the only jurisdiction to enact and then subsequently modify a universal pharmacare program for children, thereby creating the conditions conducive for a quasi-experimental policy evaluation. We were therefore interested in whether removal of insurance status as a barrier to access would promote further use of these drugs, a finding which would have potentially important clinical and health systems implications. Prior research has found that OHIP + was associated with an increase in the number of publicly covered prescriptions for medications, including those for diabetes and asthma [25].

## Methods

### Setting and study design

We conducted a population-based, cross-sectional time series analysis of antidepressant and antipsychotic units dispensed monthly by Ontario community pharmacies to children, regardless of payer, between September 1, 2014, and February 29, 2020. Because of data limitations, our analyses were restricted to children aged 18 years of age and under.

### Data sources

We used the IQVIA Geographic Prescription Monitor database, which contains data for over 83% of total prescriptions dispensed in Canada and uses patented geospatial methodology to provide projections of all prescription drug utilization at various levels of geography [26]. These data are used regularly in research evaluating drug policy changes [27–29]. This data source includes aggregate prescription units and reflects all payer types, including cash. We also used Statistics Canada’s annual population estimates for Ontario to population-adjust dispensing rates [30]. Research ethics approval was not required for this study because only aggregated, de-identified data were provided to the researchers.

### Outcomes

Our primary outcome was the monthly population-adjusted rate of antipsychotic tablets and long-acting formulations of these drugs, or antidepressant tablets, dispensed per 1,000 children and adolescents.

### Statistical analysis

We used interventional autoregressive integrated moving average (ARIMA) models to examine the association of universal drug coverage with the population-adjusted rate of antidepressant and antipsychotic units dispensed [31]. Interventional ARIMA is a regression-based method of interrupted time series analysis used to examine and quantify the effect of interventions and events on time series data while accounting for underlying trend, seasonality and autocorrelation [31]. These methods are commonly used for the evaluation of policy and public health interventions, as well as examination of sudden stressors on the health of populations [32–37]. We differenced the time series to account for temporal trends and used the augmented Dickey-Fuller test to confirm stationarity [38]. We selected model parameters using the residual autocorrelation function, partial autocorrelation function, and inverse autocorrelation function correlograms. We chose the final model using autocorrelation plots and the Ljung-Box chi-square test for white noise [31, 39]. We applied a modified step function to test for changes in the rate of psychotropic drug dispensing between January 1, 2018, and March 31, 2019, when OHIP + was implemented, and all children had universal drug coverage. We also derived expected antidepressant and antipsychotic dispensing rates for the period following the implementation of OHIP + using data between September 2004 and February 2017. These rates represent those which would have been observed in the absence of the OHIP + program. We determined the relative percent changes between the observed and predicted stimulant dispensing rates and estimated associated 95% confidence intervals using the Poisson distribution. We then applied a ramp transfer function beginning on April 1, 2019, to account for revisions to the program covering only children without private insurance.

We conducted the analyses using SAS Enterprise Guide version 9.4 (SAS Institute, Cary, NC), the SAS/ETS Time Series Forecasting System, and R Studio.

## Results

### Overall trends in antidepressants and antipsychotics dispensed

**Fig. 1:**
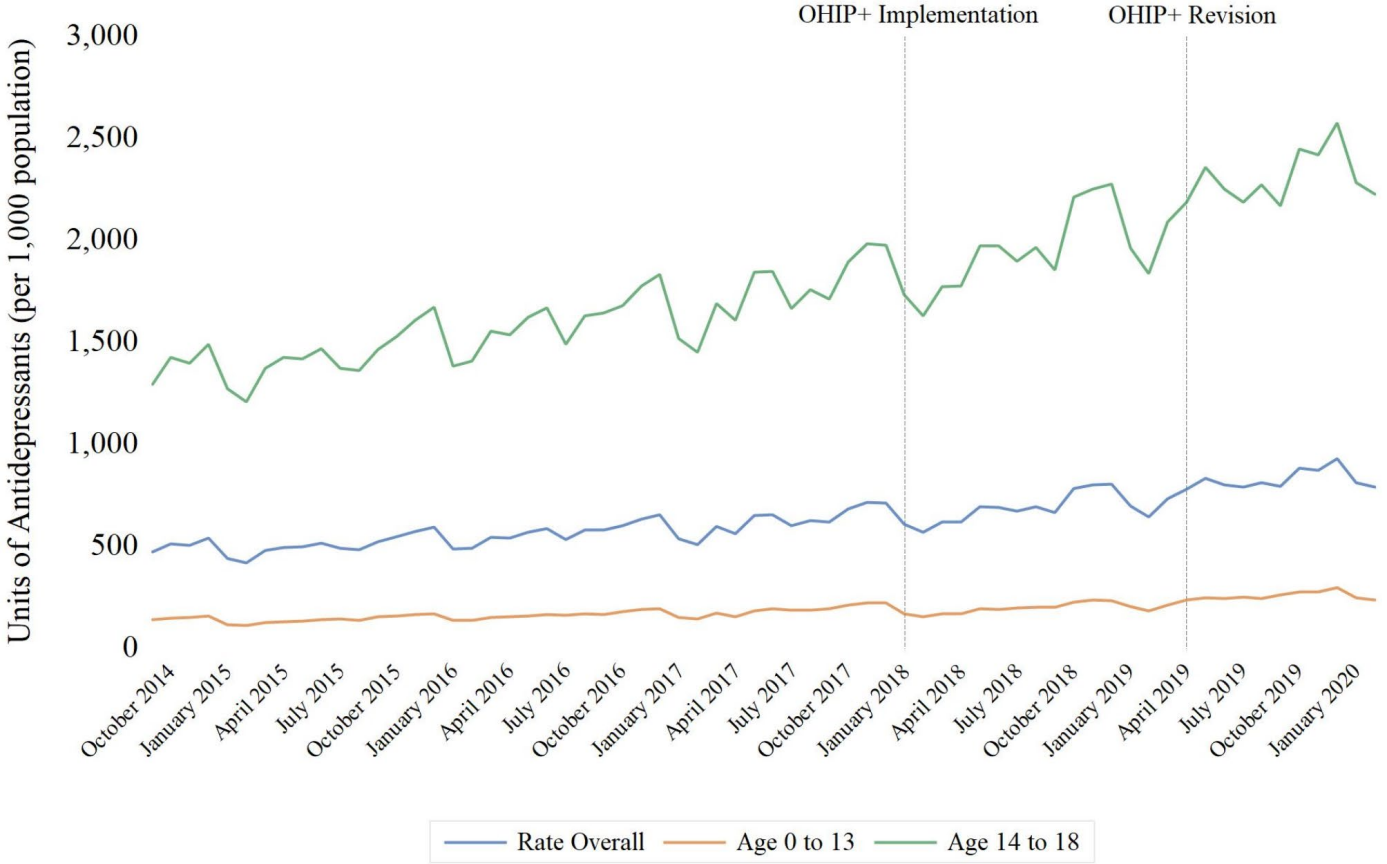
Association of OHIP + implementation (January 2018) and modification (April 2019) with monthly rates of antidepressant dispensing per 1,000 Ontario residents between the ages of 0 and 18, September 2014 to February 2020

**Fig. 2:**
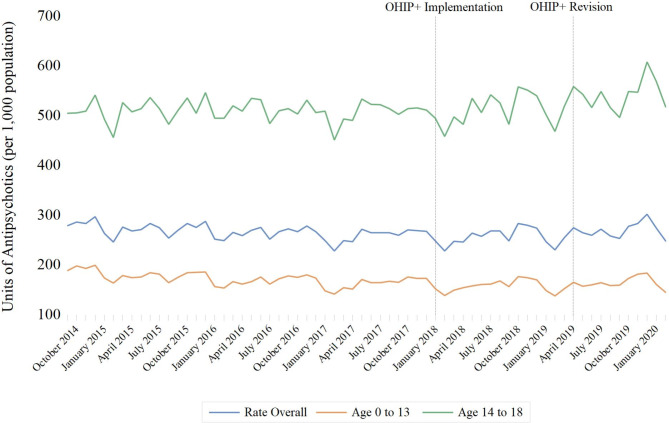
Association of OHIP + implementation (January 2018) and modification (April 2019) with monthly rates of antipsychotic dispensing per 1,000 Ontario residents between the ages of 0 and 18, September 2014 to February 2020

Between September 1, 2014, and February 29, 2020, 121 million units of antidepressants and 51 million units of antipsychotics were dispensed to children *≤* 18 years of age in Ontario. Adjustment for population size yielded rates of 7.6 antidepressant units and 3.2 antipsychotic units dispensed per resident per year, respectively. The rate of antidepressant medication dispensing increased by 67.6% (95% confidence interval [CI]: 67.2–67.9%) between September 2014 and February 2020 (from 471.7 to 790.5 units per 1,000 residents) (Fig. 1), a finding driven mainly by a 72.0% (95% CI 71.6–72.4%) increase in use (from 1,296 units to 2,228.4 units per 1,000 residents) among children aged 14 to 18 years. In contrast, the rate of antipsychotic units dispensed remained relatively stable over the study period, ranging from 228.4 to 302.3 units per 1,000 (Fig. 2). These trends differed by age stratum, with a 23.2% (95% CI 22.8–23.5%) decrease in rate among children aged ≤ 13 years (from 189.1 units per 1,000 population to 145.3 units per 1,000 population) and a 2.5% (95% CI 2.0–2.9%) increase among those aged 14 to 18 years over the study period (from 505.5 units per 1,000 population to 518.1 per 1,000 population).

### Association of universal drug coverage with dispensing

**Fig. 3:**
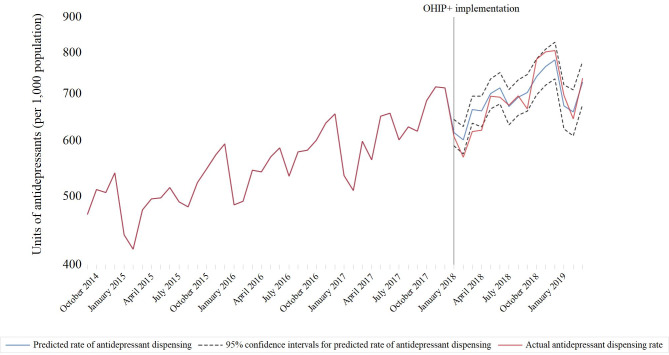
Actual versus predicted rates of antidepressant dispensing per 1,000 Ontario residents between the ages of 0 and 18 during OHIP+ (January 2018 to March 2019)

**Fig. 4:**
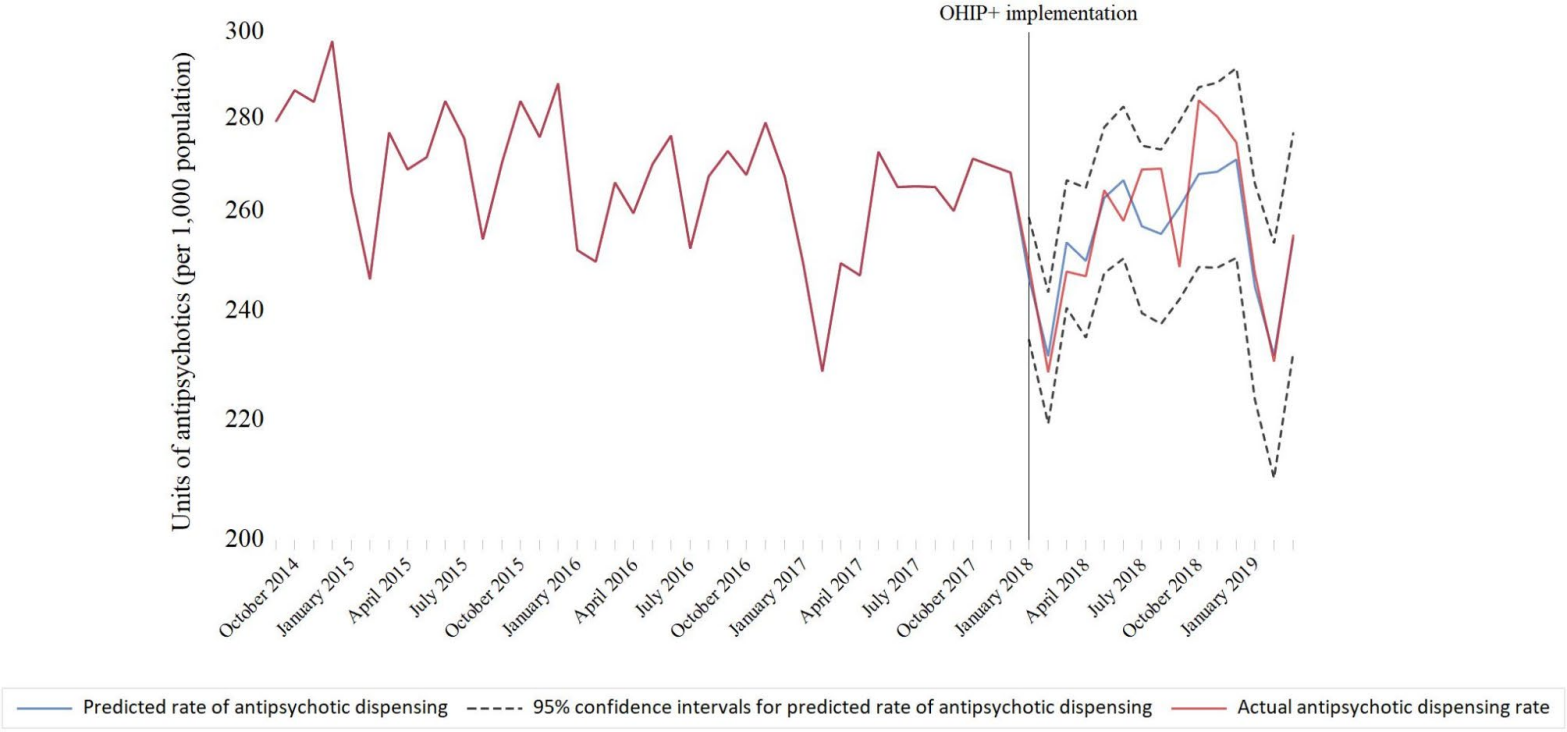
Actual versus predicted rates of antipsychotic dispensing per 1,000 Ontario residents between the ages of 0 and 18 during OHIP+ (January 2018 to March 2019)

The implementation of universal drug coverage (OHIP+) in January 2018 was not associated with the overall rate of antidepressant (-19.3 units per 1,000 population; 95% confidence interval [CI]: -41.7 to 3.1; *p* = 0.09) (Fig. 1) or antipsychotic (1.0 unit per 1,000 population; 95% CI: -5.4 to 7.5; *p* = 0.75) (Fig. 2) units dispensed (Table 1). Predicted dispensing rates were not appreciably different from actual rates (Table 2; Figs. 3 and 4). Specifically, the observed and predicted mean rates of antidepressant dispensing between January 2018 and March 2019 were 687.4 and 691.5 units per 1,000 population, with a relative percent difference between observed and predicted rates of -0.6% (95% CI -0.8% to -0.4%). Corresponding estimates for observed and predicted antipsychotic dispensing rates were 256.9 and 254.9 per 1,000 population, respectively (relative percent difference between observed and expected rates: 0.8%; 95% CI 0.5–1.1%). In addition, subsequent changes to the program limiting coverage to children without private insurance were not associated with antidepressant (0.3 units per 1,000; 95% CI: -7.4 to 7.9; *p* = 0.94) or antipsychotic (1.0 units per 1,000; 95% CI: -0.9 to 2.9; *p* = 0.29) dispensing trends. There was a small but significant decline in the rate of antidepressant units dispensed among children ≤ 13 after the initiation of OHIP+ (-22.2 units per 1,000 population (95% CI: -28.2 to -16.2; *p* < 0.001)), although trends appear to have returned to pre-OHIP + rates following modification of the program in April 2019.

**Table 1:** ARIMA model results for rate of antidepressant/antipsychotic units per 1,000 population

Rate of antidepressant units per 1,000 population	ARIMA model (no intercept)^a^	Step Intervention:^b^ Jan. 2018			Ramp intervention:^c^ Apr. 2019	
		Estimate (95% CI)		*P*-value	Estimate (95% CI)	*P*-value
**Overall**	(2,(1,12),0)	-19.3 (-41.7, 3.1)		0.09	0.3 (-7.4, 7.9)	0.94
≤ **13 years**	(3, (1,12),0)	-22.2 (-28.2, -16.2)		< 0.01	-1.0 (-3.0, 0.90)	0.30
**14–18 years**	(4,(1,12),0)	-29.6 (-82.0, 22.7)		0.27	-1.4 (-17.7, 14.8)	0.86
**Rate of antipsychotic units per 1**,**000 population**	**ARIMA model (no intercept)**	**Step Intervention: Jan. 2018**			**Ramp intervention: Apr. 2019**	
		**Estimate (95% CI)**		***P***-**value**	**Estimate (95% CI)**	***P***-**value**
**Overall**	(8,(1,12),0)	1.0 (-5.4, 7.5)		0.75	1.0 (-0.9, 2.9)	0.29
≤ **13 years**	(7,(1,12),0)	0.0 (-4.2, 4.2)		0.98	0.7 (-0.5, 1.9)	0.25
**14–18 years**	(9,(1,12),0)	3.0 (-16.8, 22.8)		0.77	2.3 (-3.7, 8.3)	0.46

^a^Model specification represented as (p, d, q): p is the number of lags of the dependent variable, representing the autoregressive nature of the model; d represents the number of times the data have to be differenced to ensure stationarity, and “1 12” represents seasonal differencing; q is the number of lags for the error term, representing the moving average part of the model

^b^Indicating whether implementation of OHIP + was associated with an immediate and sustained change in dispensing

^c^Indication whether modification of OHIP + was associated with change in slope of monthly dispensing rate

**Table 2:** Predicted and actual antidepressant and antipsychotic dispensing rates in children following the implementation of OHIP+ (January 2018 to March 2019)

Month	Predicted rate (units per 1000) of antidepressant dispensing in absence of OHIP+ (95% confidence interval)	Actual rate (units per 1000) of antidepressant dispensing following OHIP+	Predicted rate (units per 1000) of antipsychotic dispensing in absence of OHIP+ (95% confidence interval)	Actual rate (units per 1000) of antipsychotic dispensing following OHIP+
January 2018	616.8 (590.1 to 643.6)	608.7	246.7 (234.6 to 258.7)	249.0
February 2018	601.9 (574.8 to 629,1)	568.9	231.7 (219.5 to 243.8)	228.7
March 2018	665.1 (635.8 to 694.4)	618.8	253.7 (240.7 to 266.6)	247.8
April 2018	661.7 (628.5 to 694.9)	621.1	250.0 (235.1 to 264.9)	246.9
May 2018	700.4 (666.0 to 734.9)	694.1	262.8 (247.5 to 278.1)	264.5
June 2018	713.8 (677.2 to 750.3)	692.5	266.6 (250.4 to 282.7)	258.0
July 2018	671.0 (632. 4 to 709.7)	674.5	256.9 (239.7 to 274.0)	268.9
August 2018	692.5 (652.3 to 732.6)	695.0	255.4 (237.7 to 273.1)	269.0
September 2018	703.2 (661.2 to 745.1)	666.6	261.0 (242.5 to 279.5)	248.7
October 2018	741.0 (697.4 to 784.7)	784.3	267.9 (248.7 to 287.1)	284.2
November 2018	765.0 (719.9 to 810.2)	802.8	268.4 (248.7 to 288.2)	280.4
December 2018	781.8 (735.1 to 828.5)	806.5	271.0 (250.6 to 291.5)	274.7
January 2019	672.5 (624.3 to 720.7)	696.6	244.9 (223.8 to 266.0)	247.6
February 2019	659.4 (609.8 to 709.0)	644.9	231.8 (210.1 to 253.5)	230.7
March 2019	726.7 (675.7 to 777.7)	735.6	254.5 (232.2 to 276.7)	255.0

## Discussion

In this population-based study, we found increasing rates of antidepressant medication dispensing but largely stable rates of antipsychotic medication dispensing among children in Ontario, Canada, over a six-year period. However, implementing and modifying a universal drug coverage program was not significantly associated with these trends. Moreover, observed rates did not differ appreciably from those that would have been expected in the absence of OHIP+. These findings suggest that factors other than drug insurance status are the primary determinants of children’s antidepressant or antipsychotic use in Ontario.

Several mechanisms may underlie the lack of an association between universal pharmacare and the use of these drugs in children. A recent survey found that while roughly half of Canadians with mental health challenges have difficulty accessing health care services, the need for medication was the most likely to be met (85.4%) [40]. Conversely, access to non-pharmacologic therapies such as counselling was the least likely to be met. These findings suggest that available private and public drug plans may meet the pharmacological needs of most individuals with mental health conditions, including children 18 years of age and under, who are typically covered under the drug plans of their parents. Moreover, many of the drugs studied are available as relatively inexpensive generic products, the costs of which may not be prohibitive to many families. In addition, the prices for many of these products decreased over the study period because of the pan-Canadian Generic Tiered Pricing Framework starting in 2014. Furthermore, the effects of OHIP + on antidepressant and antipsychotic prescribing may have been offset by regulatory warnings alerting prescribers about drug safety issues with these drugs [41, 42] and the publication of Canadian guidelines recommending routine monitoring for the endocrinologic and cardiovascular effects of antipsychotics in children [43]. The latter point may be particularly salient for younger children and explain our finding of decreased antipsychotic medication dispensing in children under 13 years of age. However, our study was not developed to identify determinants of antipsychotic and antidepressant dispensing in children. Additional research is required to investigate factors associated with the use of these drugs in children and examine whether medication coverage is a significant factor. This may be especially salient for antipsychotics, where past research has highlighted socioeconomic gradients in the use of these drugs, with children from lower income families being more likely to be prescribed antipsychotics than those from high income families [17].

Strengths of our study include capturing prescriptions paid for by any means, including private insurance, public payer, and out-of-pocket cash payments. However, our study has limitations. First, we could not assess indications for antidepressant or antipsychotic medication dispensing. A recent study from Alberta found that most antipsychotic dispensations in children were not prescribed for psychotic symptoms, for which evidence of psychotropic medications is strongest. Instead, they were prescribed for attention-deficit/hyperactivity disorder, disruptive behaviour and nonspecific mood problems [44]. Second, we were limited by the aggregate prescription-level nature of the data. We therefore did not have clinical or diagnostic information or sociodemographic data, precluding us from examining individual-level characteristics associated with dispensing trends or determining patient diagnoses. Specifically, it would be important to stratify these findings by income to determine if the introduction of universal drug coverage increased prescribing among lower-income individuals who do not qualify for drug access through social assistance and for whom cost would have placed a higher burden on access. Third, we used unit counts as measures of antipsychotic and antidepressant utilization. However, this approach is commonly used in studies using aggregate prescription level data such as ours and has been shown to provide a good estimate of utilization [28, 45–48]. Fourth, our findings our based on data from a single Canadian province, potentially limiting the generalizability of our findings. Finally, we could not access dispensing trends in youth between 18 and 24, due to data limitations. Because these individuals may no longer be covered through their parents’ insurance plans and can access prescriptions without parental involvement or consent, it is possible that OHIP + was associated with increased use of these drugs in this population.

## Conclusion

Implementing a publicly funded pharmacare program covering medication costs for all children in Ontario was not associated with a change in antidepressant or antipsychotic dispensing. These findings imply that medication coverage may not be the primary determinants of use of these drugs in Ontario children and may allay concerns that liberalizing drug coverage would increase pediatric use of these drugs.

## Data Availability

The statements, findings, conclusions, views, and opinions contained and expressed in this study are based in part on data obtained under license from IQVIA, Canada Inc. concerning the following information service(s): IQVIA’s Geographic Prescription Monitor, data period September 1, 2014 and February 29, 2020. All rights reserved. The statements, findings, conclusions, views, and opinions expressed herein are not necessarily those of IQVIA Canada Inc. or any of its affiliated or subsidiary entities. The authors had no special access privileges and other researchers may license the data from IQVIA directly (www.iqvia.com).

## Abbreviations

ARIMA: Autoregressive integrated moving average
Cis: Confidence intervals
